# Level of asthma control and mental health of asthma patients during lockdown for COVID-19: a cross-sectional survey

**DOI:** 10.1186/s43168-021-00058-x

**Published:** 2021-02-24

**Authors:** Dina S. Sheha, Asmaa S. Abdel-Rehim, Osama M. Abdel-Latif, Maryam A. Abdelkader, Riham H. Raafat, Sarah A. Sallam, Nayera S. Mostafa

**Affiliations:** 1 Department of Internal Medicine, Allergy and Clinical Immunology, Faculty of Medicine, Ain Shams University, Cairo, Egypt; 2 Department of Chest Diseases, Faculty of Medicine, Ain Shams University, Cairo, Egypt; 3 Department of Psychiatry, Faculty of Medicine, Ain Shams University, Cairo, Egypt; 4 Department of Community, Environmental and Occupational Medicine, Faculty of Medicine, Ain Shams University, Cairo, Egypt

**Keywords:** COVID-19, Lockdown, Bronchial asthma, Disease control, Mental health, Asthma control test

## Abstract

**Background:**

Lockdown has been enforced globally to curb the spread of SARS-CoV-2. Patients with uncontrolled asthma are at risk of severe illness from COVID-19, highlighting the priority of adequate asthma control during the pandemic. Staying indoors exposes asthmatics to indoor asthma triggers, including disinfectants used for limiting the virus spread, in addition to psychological stresses of the pandemic which represent crucial contributors to loss of asthma control. Elective medical care, curtailed by the lockdown procedures, compromises adequate asthma follow up. The current study evaluated the effect of COVID-19 lockdown on the level of asthma control and mental health of bronchial asthma patients. The study included 264 bronchial asthma patients, aged 12 years and older, who responded to an online questionnaire including the asthma control test to evaluate asthma control in the preceding 4 weeks. Anxiety and depression scores and the impact of event scale were also provided.

**Results:**

Seventy percent of asthmatics had uncontrolled asthma, and disinfectant use was associated with perceived increase in asthma symptoms in 77.7%. Anxiety and depression were associated with uncontrolled asthma in 50% of participants, suggesting a possible psychological impact on asthma patients.

**Conclusions:**

During lockdown, asthma patients participating in the study had significantly uncontrolled disease and associated anxiety and depression. Since regular follow-up of asthma patients is cornerstone to adequate asthma control, alternative methods of medical care for asthma patients during lockdown are warranted, and particular need for mental health support ought to be provided as a continuum to adequate asthma control.

## Background

The novel coronavirus named severe acute respiratory syndrome coronavirus-2 (SARS-CoV-2) emerged at the end of December 2019. Coronavirus disease 2019 (COVID-19) is the clinical syndrome associated with SARS-CoV-2 infection. It is characterized by a respiratory syndrome with varying degrees of severity, ranging from mild upper respiratory tract illness to interstitial pneumonia and acute respiratory distress syndrome (ARDS) [1]. Since the World Health Organization (WHO) declared COVID-19 a pandemic in early March 2020, the WHO and the Center for Disease Control and Prevention (CDC) have been considered the most reliable sources of information for the COVID-19 global pandemic. The CDC listed moderate to severe asthma, especially if not well controlled, as high-risk for severe illness from COVID-19 [2].

Asthma is a heterogeneous chronic respiratory disease that affects around one to 18% of the population worldwide. Interim guidance on asthma management during COVID-19 pandemic that was added in the most updated version of Global Initiative for Asthma (GINA) guidelines pointed out the importance of maintaining proper asthma control during the epidemic, and urged asthma patients to follow their written action plan in case of worsening asthma symptoms [3]. Achieving good asthma control is considered critical in minimizing future risk of asthma-related mortality and exacerbations.

Simultaneously, partial lockdown due to COVID-19 outbreak was imposed in a majority of countries around the world. Large-scale home confinement has significantly compromised direct patient encounters in clinical facilities, and thus opportunities to objectively evaluate patients’ symptoms and provide support for patients’ psychosocial needs have diminished [4].

Asthma patient-health professional partnership is paramount in ensuring effective asthma control. Regular review of asthma patients by a health care provider or a trained healthcare worker is an essential component of effective asthma management [3], and regular monitoring for treatment adjustment is unattainable during lockdown. Outbreak of COVID-19 can be stressful for asthma patients, especially with CDC warnings that patients with chronic illness are at higher risk for severe illness from COVID-19 [2]. Hence, we speculate that asthma patients are likely to respond more strongly to stress of the crisis. Disrupted medical care due to medical facility closures and service reduction during lockdown curbs patients’ access to medical services for reassurance, psychosocial monitoring, and treatment adjustment. Additionally, staying indoors for patients with asthma poses an important contributing factor to loss of control of their disease. An important contributor to asthma control depends on environmental factors, and numerous indoor inhalant allergens are associated with increased risk of asthma exacerbations, including house dust mites, animal dander, cockroaches, and mold [5].

Moreover, CDC recommendations for disinfection of households to limit spread of SARS-CoV-2 advises using volatile compounds such as 70% alcohol and diluted household bleach solutions which might worsen pre-existing asthma, and numerous studies have suggested worsening asthma symptoms in relation to the use of household cleaning products [6]. Hence, CDC released detailed recommendations advising specific precautions for use of disinfectants by asthma patients during the pandemic [7].

Among the psychosocial effects of quarantine previously studied are depression, anxiety, psychosomatic preoccupations, and insomnia, and among people at heightened risk of psychosocial effects of pandemics are people with preexisting medical conditions [8]. Despite extensive evidence available on co-existing mental health disorders in asthma patients, during asthma patient care, psychological comorbidities are frequently overlooked. Nonetheless, asthma and anxiety are highly co-morbid, and their interaction leads to exacerbation of both conditions [9]. However, it is unclear whether they are interdependent or if one disorder precedes the other. A study reported prevalence of anxiety and depression on outpatients with asthma of 36.9% and 11% respectively. Anxiety and depression were associated with poor asthma control and lower asthma control test (ACT) scores [10].

Thus, it is of timely importance to evaluate the level of control and mental health of asthma patients during the lockdown enforced due to the respiratory-virus driven pandemic. Psychosocial problems represent an important modifiable risk factor for asthma exacerbations [3], and regular follow-up is significantly halted by the lockdown. Hence, we sought to identify asthma control and mental health of asthma patients during lockdown for COVID-19 that was imposed in Egypt in mid-March, in order to identify verifiable causes for lack of asthma control and record mental health issues that patients withstood during lockdown.

## Methods

The current study is a cross-sectional, observational, and analytical study. Due to the current circumstances of COVID-19, and lack of elective medical services and outpatient clinics, the study was conducted through an online questionnaire sent through social media to asthma patients who were regularly followed up by co-authors of the current study at either the Pulmonology, or Allergy and Immunology clinic at Ain Shams University hospitals. We included 264 bronchial asthma patients aged 12 years and older who were diagnosed with asthma for at least 6 months prior. The sample size was calculated based on a proportion of 22% of combination of depression and anxiety during COVID-19 pandemic in a study done by Gao and co-workers [11], with a 5% margin of error 95% confidence interval. A convenience non-random sampling was used for recruitment.

Patients with confirmed COVID-19 infection, patients with cardiac disease, patients with documented history of psychological disorders including anxiety and depression, and patients receiving systemic steroids or antipsychotic medications were excluded from the study. All patients fulfilling the inclusion criteria were included in the study up until the completion of the sample size. Asthma was defined by self-reporting of a previous diagnosis of asthma by a physician. Children were allowed to be assisted by their parents or care-givers for filling the questionnaires.

The online questionnaire included:

1. Personal and medical data of the patients (age, gender, associated allergy, smoking status etc.)
2. Risk factors and triggers for bronchial asthma (house dust, mold, pets etc.)
3. An Arabic version of the validated asthma control test (ACT) [12–15] translated by the Health Authority of United Arab of Emirates. It consists of five questions, each scored one to five. The total score denotes the level of asthma control as follows; 19 or less denotes that asthma is not well-controlled or poorly controlled, 20–24 denotes that asthma is controlled and 25 denotes that asthma is completely controlled
4. An Arabic version of Hospital anxiety and depression scale (HADS). It includes 14 items assessing anxiety (seven-item) and depression (seven-item), which are rated on a four point Likert-type (from zero to three). The scores in each subscale are computed by summing the corresponding items, with maximum scores of 21 for each subscale. A score of zero–seven is considered as normal, eight–ten as a borderline case, and 11–21 as a case [16]
5. An Arabic version of the Impact of Events Scale-Revised (IES-R). It includes 22 items assessing the core symptom cluster of post-traumatic stress disorder (PTSD): avoidance (eight items), intrusion (eight items), and hyper arousal (six items). Each item is scored on a five-point Likert scale (ranging from zero = not at all to four = extremely). The higher the score, the greater the concern for PTSD [17]. The impact of event scale is widely used for assessment of the impact of traumatic life events and is concerned with measuring event-specific distress by evaluating stress-related thoughts and behaviors that patients experience [18]

Statistical analysis was performed using SPSS statistical package version 21. Qualitative data are presented as frequency and percentages. Quantitative variables are presented as Mean ± Standard deviation (SD). Chi-squared test was used to test the relation between mental health status and ACT. Associations between quantitative variables were tested using Pearson correlation coefficients. Significant difference was considered at a *P* value < 0.05.

### Ethical consideration

Ethical approval was obtained from Faculty of Medicine, Ain Shams University Ethics Committee, Cairo, Egypt (FMASU 19/2020). Confidentiality of data was ensured to the study team. Informed written consent was obtained from patients 18 years or older following the provision of an explanation of the study rationale and procedures, and an assent was obtained from patients 12–18 years old, as well as written consent from their guardians.

## Results

The study included 264 participants, approximately 60% were females, and age ranged from 12 to 78 years; 42.4% of the study participants are non-working, including housewives, students, or retired persons. Further, 48.5% have professional jobs, while 9.1% are manual workers. Personal and medical data are displayed in Table 1.

**Table 1:** Personal and medical data of the study participants

Item	*N* (%)
Age (Mean ± SD)	36.05 ± 12.9 (range 12–78 years)
Gender	
Male	106 (40.2)
Female	158 (59.8)
Comorbidities	
Nasal allergy	176 (66.7)
Food allergy	33 (12.5)
Diabetes	21 (8)
Hypertension	19 (7.2)
Medication allergy	17 (6.4)
Others	33 (12.5)
Smoking	
Current smoker	21 (8)
Former smoker	35 (13.3)
Never smoked	208 (78.8)
Exposure to passive smoke	85 (32.2)
Exposure to home allergen	150 (56.8)
Pet exposure at home	58 (22)
Mold exposure at home	24 (9.1)
Mold exposure at home	24 (9.1)
Pattern of symptoms	
Seasonal	124 (47)
All year long	43 (16.3)
All year long, with seasonal worsening	97 (36.7)
Frequency of symptoms	
Twice or less weekly	90 (34.1)
3–6 times weekly	56 (21.2)
Once a day	47 (17.8)
Throughout the day	71 (26.9)
Worsened symptoms	
Outdoors	37 (14)
Indoors	70 (26.5)
No difference	157 (59.5)
Presenting symptoms	
Shortness of breath	190 (72)
Wheezes	103 (39)
Chronic cough	80(30.3)
Chest tightness	68 (25.8)
Asthma control test	
Poorly controlled	183 (69.3)
Controlled	67 (25.4)
Completely controlled	14 (5.3)
Duration of bronchial asthma illness (mean ± SD in years)	12.66 ± 11.78 (0.1–58)

As shown in Table 1, 47% of the study participants have seasonal asthma. Shortness of breath is the most common presenting symptom in 72%, followed by wheeze, chronic cough, and chest tightness. According to ACT score, about 70% of the study participants have poorly controlled asthma. Then, 150/264 (56.8%) of the study participants perceived various indoor factors as a triggers for asthma symptoms, and among perceived triggers were pets (cats, dogs, hamsters, and birds), molds, strong smells, house dust, cleaning and disinfection agents, and tobacco smoke. Most of those participants had poorly controlled asthma, but with no statistical difference when compared with controlled and completely controlled asthma patients.

Table 2 shows that 46% of the study participants experienced increased symptoms during lockdown, and almost 90% reported current disinfectants use; 78% stated that their asthma symptoms increased in direct relation to disinfectant use.

**Table 2:** Characteristics of the study participants during lockdown

Characteristic	*N* (%)
Increased Symptoms during lockdown	121 (45.8)
Shortness of breath	96 (36.4)
Wheezes	44 (16.7)
Cough	34 (12.9)
Chest tightness	37 (14)
Disinfectants use	236 (89.4)
Chlorine bleach	198 (75)
Alcohol 70%	124 (47)
Other	35 (13.3)
Frequency of disinfectants use	
More than once daily	37 (15.4)
Once daily	87 (36.1)
More than once weekly	58 (24.1)
Once weekly	49 (20.3)
Less than weekly	10 (4.1)
Increased symptoms due to disinfectants use	192 (77.7)
Shortness of breath	131 (49.6)
Wheezes	49 (18.6)
Chronic cough	72 (27.3)
Chest tightness	73 (27.7)

**Fig. 1:**
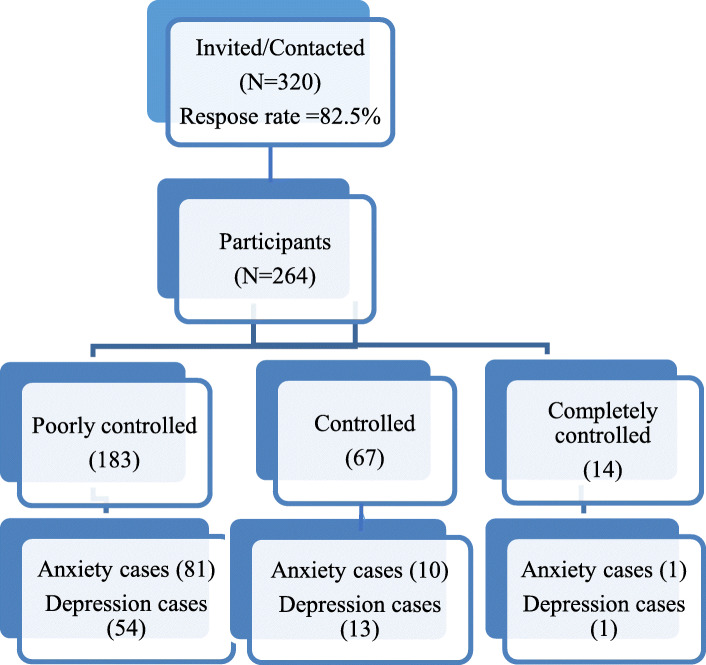
Flowchart of study participants according to ACT and HADS

Figure 1 shows the classification of the study participants according to ACT score, and the number of anxiety and depression cases in each ACT category. About 35% and 26% of the study participants were anxiety and depression cases respectively as shown in Table 3. The mean of IES-R equals to 35.91 ± 19.09.

**Table 3:** Psychological morbidities of the study participants

Test	*N* (%)
Anxiety	
Normal	123 (46.6)
Borderline	49 (18.6)
Case	92 (34.8)
Depression	
Normal	121 (45.8)
Borderline	75 (28.4)
Case	68 (25.8)
	Mean ± SD
Impact of event scale (IES-R) total	35.91 ± 19.09
IES-R Intrusions	12.00 ± 7.50
IES-R Avoidance	12.21 ± 7.66
IES-R Hyperarousal	11.71 ± 5.80

A statistically significant association was found between ACT and HADS as shown in Table 4. A one-way analysis of variance (ANOVA) test showed that there is a significant difference of ACT based on patient’s depression and anxiety scores (*p* = 0.009 and ˂ 0.001 respectively). Post-hoc comparison to evaluate pairwise difference among group means was conducted using Tukey test. The test revealed significant pairwise difference between the mean scores of depression cases or patients with normal depression score (*p* ˂ 0.05). Patients with borderline depression do not significantly differ from the other two groups. Regarding anxiety, significant pairwise difference between the three groups (normal, borderline, and cases) was found. Student *t* and ANOVA tests showed no significant difference between means of ACT based on socio-demographic data (age, gender, occupation, residence).

**Table 4:** Association between ACT and HADS

	Asthma control			Total	Fisher exact test	*P* value
	Poorly controlled	Controlled	Completely controlled			
Anxiety					26.421	˂ 0.001*
Normal	71 (38.8%)	41 (61.2%)	11 (78.6%)	123		
Borderline	31 (16.9%)	16 (23.9%)	2 (14.3%)	49		
Case	81 (44.3%)	10 (14.9%)	1 (7.1%)	92		
Depression					9.680	0.041*
Normal	74 (40.4%)	36 (53.7%)	11 (78.6%)	121		
Borderline	55 (30.1%)	18 (26.9%)	2 (14.3%)	75		
Case	54 (29.5%)	13 (19.4%)	1 (7.1%)	68		

**Fig. 2:**
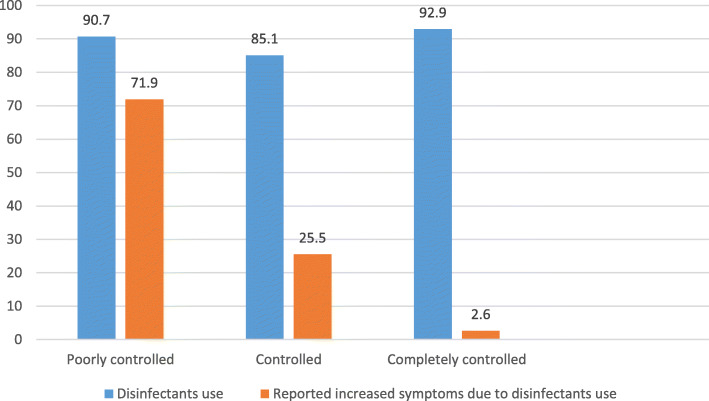
Disinfectant use and perceived increased symptoms distributed among participants

Figure 2 shows the perceived increase in symptoms and disinfectant use among participants. Perceived symptoms were compared according to the level of asthma control. Approximately 72% of patients who perceived increased symptoms were poorly controlled, compared to 25% controlled and 3% completely controlled patients (*p* value 0.002).

An inverse correlation was found between ACT and psychological tests with statistical significance as shown in Table 5.

**Table 5:** Correlation between psychological tests and asthma control test

	Asthma control test	
	Correlation *r*	*P* value
Anxiety scale	− 0.366	˂ 0.001
Depression scale	− 0.198	0.001
IES-R total	− 0.196	0.001
IES-R Intrusions	− 0.162	0.008
IES-R Avoidance	− 0.135	0.028
IES-R Hyperarousal	0.257	˂ 0.001

*IES*-*R* impact of event scale

Table 6 shows the effect of personal and psychological factors on ACT according to multiple linear regression models, anxiety is negatively associated with ACT (β = − 0.348, 95% CI: 0.495, − 0.201, *p* = 0.000).

**Table 6:** Regression analysis of the effect of personal and psychological factors on ACT (*N* = 264)

Independent variables	Unstandardized coefficients		Standardized coefficients	*t*	Sig.	95% Confidence interval		*R*^2^	*p* value of F statistics
	B	Std. error	Beta			Lower bound	Upper bound		
(Constant)	21.373	1.476		14.481	0.000	18.467	24.279	0.145	.000
Age	0.008	0.022	0.022	0.379	0.705	− 0.035	0.052		
Gender	− 0.994	0.578	− 0.102	− 1.720	0.087	− 2.133	0.144		
Anxiety	− 0.348	0.075	− 0.353	− 4.664	**0**.**000**	− 0.495	− 0.201		
Depression	− 0.027	0.079	− 0.023	− 0.335	0.738	− 0.182	0.129		
IER	− 0.007	0.017	− 0.029	− 0.442	− 0.442	− 0.040	0.026		

## Discussion

Enforcing global lockdown in an attempt to halt the spread of COVID-19 is not without drawbacks for asthma patients for various factors. Indoor asthma triggers are a substantial contributor to loss of asthma control, and extensive disinfectants used for cleaning surfaces can irritate the airways of asthmatics. Additionally, a mental health crisis is brewing in consequence to the COVID-19 pandemic [19], and asthma patients are specifically vulnerable since the CDC warns that “patients with moderate to severe asthma may be at a higher risk of getting very sick from COVID-19” [3]. Nonetheless, some evidence suggests that patients with asthma are not at increased risk for COVID-19 morbidity nor mortality [20], although the fear from COVID-19 infection is indeed impacting people with asthma [21, 22].

Hence, in the current study, we aimed to evaluate the level of asthma control in adolescent and adult bronchial asthma patients, and study the correlation between asthma control and mental health effects (impact of event, anxiety, and depression) during lockdown for COVID-19. Asthma control of the study participants was evaluated using the ACT score, which is a valid measure of asthma control and ongoing symptomatology [23]. The current study recorded poorly controlled asthma in 69.3% of participants, and 45.8% of participants reported increase in asthma symptoms during lockdown. The symptom that was most experienced by patients was shortness of breath in 96/264 (36.4%) participant, while wheeze, chest tightness, and cough were reported by 16.7%, 14% and 12.9% of participants respectively. Although indoor inhalant allergens, including animal dander, cockroach, and molds, are common triggers for asthma symptoms and exacerbations [5], we did not find a statistically significant association between asthma control by ACT score and indoor allergens, whether pets (animal dander) or indoor molds.

Subsequently, we sought to determine the association between staying indoors and disinfectant use, and the level of control of bronchial asthma. Extensive disinfectant use was reported by 89.4% of the study participants, and increase in asthma symptoms after disinfectant use was reported by 81.4% of those who used disinfectants. The most frequent symptom reported was shortness of breath (49.6%); chest tightness, cough, and wheeze were reported by 27.7%, 27.35, and 18.6% of patients respectively. Among poorly controlled asthma patients, 71.9% reported increased asthma symptoms after disinfectant use, compared to 25.5% in controlled group and 2.6% in the completely controlled group, with statistical significance (*p* = 0.002).

The irritant effects of cleaning products on the respiratory system is subject of growing attention [6], as the chemicals contained in cleaning products are thought to be a contributor to both the development of asthma and to symptom exacerbation among asthmatics. Among sensitizers in cleaning agents are disinfectants and quaternary ammonium compounds. Bleach (sodium hypochlorite) in cleaning products is a robust airway irritant [24]; however, it is the main disinfectant recommended by the WHO for cleaning environmental surfaces in context of COVID-19 [25]. Another proposed mechanism of airway irritation induced by disinfectants hypothesizes true sensitization induced by an immunological mechanism, through inciting damage of the bronchial epithelium, and eliciting a pro-inflammatory response via neurogenic inflammation of exposed nerve endings. This is followed by altered lung permeability that finally leads to airway epithelium remodeling [26].

In terms of mental health, the main psychological impact of COVID-19 pandemic to date is elevated levels of stress or anxiety. However, as new measures are introduced—particularly quarantine and its effects on people’s usual activities and routines—levels of loneliness and depression are on the rise [27]. A Medline search revealed that most research on COVID-19 mental health effects has been targeting vulnerable groups especially healthcare workers on the frontline. Levels of stress among 1257 Chinese healthcare workers in a JAMA network publication reported anxiety in 44.6% and depression in 50.4% [27].

The interplay between asthma and anxiety has been extensively studied [8, 28]. Anxiety causes hyperventilation, which causes bronchoconstriction, thereby exacerbating asthma symptoms [29]. Moreover, airway inflammation is triggered by cytokines and neuropeptides release during an anxiety episode [30]. Psychosocial stressors have been reported as both inciting and precipitating factors for asthma [31]. Anxiety compromises good asthma care and medication adherence leading to a self-perpetuating feedback cycle [8]. On assessing psychological morbidities of the study participants collectively, 46.6% had normal anxiety levels, in contrast to 18.6% participant with borderline anxiety, and 34.8% classified as anxiety cases.

Studying the association between level of control of bronchial asthma and psychological factors revealed that borderline anxiety and anxiety cases was recorded in 16.9% and 44.3% of participants with poorly controlled asthma respectively, with high statistical significance. In comparison with the MERS epidemic in 2012, anxiety symptoms were found in 7.6%, which is much lower than that recorded for SARS-CoV-2 [32]. However, anxiety symptoms in the aforementioned study were recorded in a normal population not asthmatics, which could account for the discrepancy in anxiety rates.

Regarding depression, 30.1% of poorly controlled asthma participants experienced borderline depression, while 29.5% were depression cases with statistical significance. Asthma control was inversely correlated with HADS and IES, suggesting that patients with controlled asthma were less likely to suffer from anxiety and depression.

In line with our results, Cooper and colleagues conducted a case-control study to compare anxiety and panic fear in adult asthma patients in a hospital setting, and found prevalence of anxiety and depression among adult asthma patients was 47.3% and 22.3% respectively [33]. Authors reported a statistically significant difference in anxiety score considering the level of asthma control, and the same with depression score. Additionally, Lu et al. estimated a 27% prevalence of depressive symptoms in adolescent asthma patients, suggesting that asthma could be considered as a risk factor for depressive symptoms in adolescents, since asthma affects daily functions requiring physical activity such as ambulation, mobility and school activities [34].

A study by Ciprandi et al. on 262 outpatients with asthma found that more than one-third of patients had anxiety and 11% had depression, and both anxiety and depression were associated with poor asthma control by ACT, and they concluded that anxiety and depression were a common comorbidity in asthma patients [10]. Emotional disorders in outpatients with asthma thus had clinical relevance. Mcdonald et al. [35] similarly reported anxiety and depression in 38% and 25% of patients with severe asthma respectively. Depression was recognized in their study as one of the treatable traits significantly associated with increased risk of asthma exacerbations over time with incident rate ratio 1.63.

Although asthma, depression, and anxiety are closely related, little is known about their causal relationship, and whether asthma coincidentally occurs with anxiety and depression, or one disease precedes the other [36]. Asthma and depression are thought to share a common mechanism. According to the hygiene hypothesis, the limited exposure to bacteria in industrialized societies causes alterations in the microbiota leading to exaggerated immune response thus increasing risk of allergic diseases and psychiatric disorders [37].

IES helped predict the post-traumatic stress symptoms in patients during and after traumatic life events including all its subscales intrusions, avoidance, and hyperarousal symptoms with a mean score 12, 12.21, 11.71 respectively, and were negatively correlated with asthma control. This could be explained by mental health analysis of high-risk populations by Mazza and colleagues, who found that patients with chronic illnesses were more susceptible to psychological distresses since they perceive themselves with poor health and thus become more vulnerable [38].

Additionally, our study is in concordance a study conducted in China by Wang et al. in 2020 [39] during the initial state of the virus spread using the same scale. Further, 53.8% of the participants had moderate or severe psychological impact and 21.7% reported mild impact. On the other hand, Zhang et al. reported a lower overall mean IES score in their study participants (13.6%), indicating a less stressful impact [40]. The discrepancy between the two studies could possibly be related to the differences in sample size and the limited scope of the latter study.

We also concur with González-Freire et al. who recently advised routine screening of anxiety, depression, and level of asthma control in asthma patients, hypothesizing that this could possibly improve quality of life of asthma patients [41]. This is particularly crucial during lockdown. Reaching out to asthma patients to ensure adequate asthma control and provide reassurance regarding mental health issues through alternative methods such as telehealth for instance, might represent proxy to regular clinic visits during lockdown.

## Conclusion

In conclusion, we hereby report that more than two-thirds of the current study participants had poorly controlled asthma during the lockdown period, and asthma symptoms worsened in relation to use of household disinfectants. Borderline and true anxiety cases and borderline and established depression affected almost half the study participants. It is necessary to emphasize the importance of detecting anxiety and depression and post-traumatic symptoms early among individuals with asthma to be able to develop suitable and timely psychological interventions. The current study draws attention to the possible effect of mental health on the control of asthma, and recommends the need for integrating mental health care in routine asthma follow up and management plan by health care professionals. Regarding disinfectants, education of asthmatics about their safe use, as well as clear warnings about extensive use in asthma patients are warranted. Developing a single validated measure of irritating levels of cleaning products in households would be of paramount importance.

Finally, it is essential to take into account our study limitations. First, past records of the study participants were not available; therefore, comparing levels of control of asthma before and during lockdown was not feasible. The study depends on participants’ subjective reporting of the symptoms constrained by the current situation which doesn’t allow clinical evaluation of the patients by means of clinical examination and required investigations. It could also be argued that social desirability bias was introduced in the survey questions regarding disinfectants use as proved by Jaén and Dalton [42], where the authors found that expectations provoked by smelling a perceived strong odor objectively influenced asthma exacerbations. The cross-sectional nature of the study does not allow for the prediction of causality, and a control group would have allowed for better characterization of effects of lockdown more generally. The study lacks longitudinal follow up, and further assessment using a comparable population after the pandemic finally clears could further ascertain this effect. Additionally, the results cannot be generalized as it included patients of average educational and economic background because of the nature of data collection. Larger-scale future studies that prospectively evaluate the effect of treatment of anxiety and depression on disease control and quality of life of asthma patients are warranted, especially taking into consideration that asthma patients are possibly a vulnerable population in the circumstances of COVID-19 pandemic.

## Data Availability

Not applicable.

## Abbreviations

COVID-19: Corona virus disease 2019
SARS-CoV-2: Severe acute respiratory syndrome corona virus 2
WHO: World Health Organization
ARDS: Acute respiratory distress syndrome
GINA: Global Initiative for Asthma
CDC: Center of Disease Control
ACT: Asthma control test
HADS: Hospital Anxiety Depression Scale
IES-R: Impact of Event Scale–Revised
PTSD: Post-traumatic stress disorder
